# Optimization of Fluoride Adsorption on Acid Modified Bentonite Clay Using Fixed-Bed Column by Response Surface Method

**DOI:** 10.3390/molecules26237112

**Published:** 2021-11-24

**Authors:** Adane Woldemedhin Kalsido, Beteley Tekola Meshesha, Beshah M. Behailu, Esayas Alemayehu

**Affiliations:** 1 Africa Centre of Excellence for Water Management, Addis Ababa University, Addis Ababa P.O. Box 1176, Ethiopia; beteley.tekola@aau.edu.et (B.T.M.); esayas16@yahoo.com (E.A.); 2 College of Engineering and Technology, Wachemo University, Hossana P.O. Box 467, Ethiopia; 3 School of Chemical and Bio Engineering, Addis Ababa University, Addis Ababa P.O. Box 1176, Ethiopia; 4 Water Development Commission, Ministry of Water, Irrigation and Energy, Addis Ababa P.O. Box 13/1067, Ethiopia; beshahnb@gmail.com; 5 Jimma Institute of Technology, Faculty of Civil & Environmental Engineering, Jimma University, Jimma P.O. Box 378, Ethiopia

**Keywords:** adsorption, fluoride remediation, acid-treated bentonite clay, fixed-bed column, response surface methodology

## Abstract

Using small-scale batch tests, various researchers investigated the adsorptive removal of fluoride using low-cost clay minerals, such as Bentonite. In this study, Column adsorption studies were used to investigate the removal of fluoride from aqueous solution using acid-treated Bentonite (ATB). The effects of initial fluoride concentration, flow rates, and bed depth on fluoride removal efficiency (R) and adsorption capability (qe) in continuous settings were investigated, and the optimal operating condition was determined using central composite design (CCD). The model’s suitability was determined by examining the relationship between experimental and expected response values. The analysis of variance was used to determine the importance of independent variables and their interactions. The optimal values were determined as the initial concentration of 5.51 mg/L, volumetric flow rate of 17.2 mL/min and adsorbent packed-bed depth of 8.88 cm, with % removal of 100, adsorptive capacity of 2.46 mg/g and desirability of 1.0. This output reveals that an acid activation of Bentonite has made the adsorbent successful for field application.

## 1. Introduction

The United Nations Development Program has set a target of delivering wholesome or healthy water for drinking to all by 2030 as a sustainable development goal. In recent years, water pollution has become a big problem all over the world. The most significant problem with groundwater quality is fluoride pollution. Mostly, as consequence of natural and human activity causes, fluoride contamination of groundwater has already become a serious problem, posing health risks to humans [1,2,3,4]. Ethiopia, as with other developing countries, is dealing with fluoride contamination, especially in the Central Rift Valley. It is estimated that over 11 million people in Ethiopia’s Great Rift Valley depend on groundwater from sources that are contaminated with high fluoride concentration [5]. Coagulation and precipitation, membrane filtration, adsorption, advanced oxidation microfiltration, and ultrafiltration methods have all been used to treat fluoride contaminated potable water. Adsorption and surface precipitation are promising water treatment methods because of their efficiency, convenience, and cost-effectiveness. They can also be used to eliminate a variety of pollutants [6,7,8].

Adsorbents derived from natural materials are ideal because of their cheap price, easy availability, and additional financial advantage of avoiding the import of advanced chemicals and products from several other areas. As a result, clay minerals such as Bentonite, Montmorillonite and Kaolinite in raw or modified forms are being studied as adsorbent material in a wide range of aspects due to their high cation-exchange potential values, permeability, large surface area, and strong adsorption capacities [9]. Numerous studies have continued to activate raw clays chemically (using bases, acids, and salts), thermally or mechanically to improve their adsorption performance.

Thermochemical treatment of clays as Bentonite using hydrochloric acid (HCl), sulfuric acid (H_2_SO_4_), and nitric acid (HNO_3_)is now becoming increasingly common as a means of improving its surface and chemical properties [10,11]. As the previous researcher, Eren and Afsin [12] reveled that structural improvements and slight decomposition of the materials caused differences in surface area between 0.25–0.4 M concentrations of HCl. As the concentration of hydrochloric acid rose, the surface area enhanced where 837.11 m^2^/g was obtained using 0.4 M HCl. Activation at a larger concentration (0.6 M), on the other hand, resulted in a decrease in the surface area. As a result, it could be an excellent option for low-cost fluoride sorbents growth. The physico–chemical properties of acid-treated bentonite clay (ATB) and its adsorption performance can be assessed using X-ray diffraction (XRD), Fourier transform infrared spectroscopy (FT-IR), Electro-diffraction X-ray (EDX), and scanning electron microscopy (SEM) and reported on our recently published paper [13].

When contemplating future practical applications of adsorbents, forecasting the lifespan of the adsorbent and its scalability is important. According to researchers, full-scale field research is the only accurate way to forecast the long-term performance of water purifying media [7]. Alternatively, the adsorbent durability can be investigated in depth using laboratory-based fixed-bed column setups. The conventional approach of optimizing several parameters generally requires a large number of tests and requires a longer time to determine the optimum amount of each variable [14]. The effect of independent factors and their interactions on the adsorption performance in continuous settings can be achieved using range of mathematical experimental design methods such as response surface methodology (RSM) [15]. RSM with central composite design (CCD) is being applied in a range of research applications, such as removal of heavy metals (mercury, cadmium, etc.), and anion removal (fluoride), [15,16,17,18,19,20,21,22,23]. Hence this work aims to investigate and optimize the fluoride adsorption performance of ATB in fixed-bed column using RSM. In the study, ATB’s capacity to eliminate fluoride was tested under varying of operating conditions (initial fluoride concentration, volumetric flow rates, and sorbent packed-bed depth) and results are reported accordingly.

## 2. Materials and Methods

### 2.1. Reagents and Chemicals

The raw Bentonite was obtained from market and the chemicals and reagents used in the analysis are SPANDS reagent, zirconium chloride, sodium arsenate, hydrochloric acid (37%), and sodium hydroxide (NaOH) supplied by Merck S.P.L., Worli, Mumbai, India-40001. All the chemicals employed were analytical grade. The Design-expert software version 7.1.5 provided by State-Ease company in Newcastle, England was applied.

### 2.2. Preparation of Sorbent

Acid-treated Bentonite adsorbent production and experimental analysis phases of the experiment were separated into two sections as per methods described in [13]. The activation process involves adding 37% HCl to Bentonite clay, followed by characterization, optimization, and final treatment under ideal conditions. The raw clay was primarily washed with ultra-pure (RO) water and calcined at 300 °C oven for 2 h to remove certain impurities. Then the calcined Bentonite (150 g) was soaked into 0.4 M HCl and continuously shaken for 2 h at 60 °C. The sample was then filtered out from the solution and cleaned with purified water multiple times until the pH of filtrate is 6–8, which is suitable for drinking water. The ATB was then dried at 105 °C for 24 h in an oven, grinded to obtain 0.212–2 mm particle size and stored in an airtight polyethylene bag. The ATB was wetted with distilled water prior to adsorption experiment in fixed-bed column to allow trapped air between the adsorbent particles to pass downward.

### 2.3. Fluoride Stock Solution Preparation

220 mg sodium fluoride (NaF) was dissolved in purified water to make a fluoride stock solution of 100 mg/L in a 1 L volumetric flask. Fluoride solution with different concentration (0.1–25 mg/L) was obtained by diluting the stock solution.

### 2.4. Fluoride Adsorption Studies

**Figure 1:**
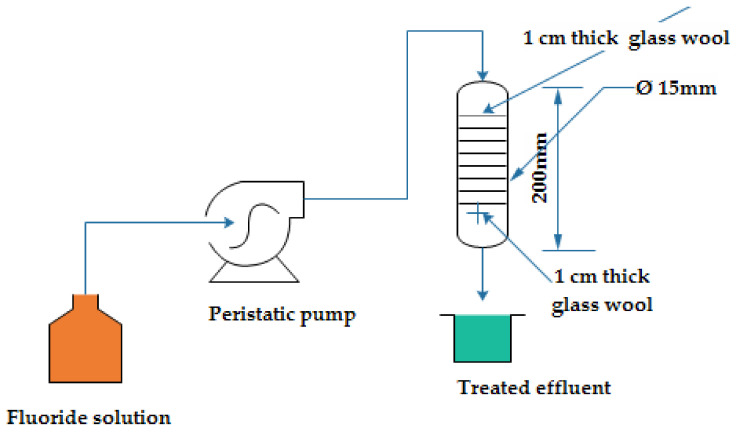
Laboratory-based diagram of the column setup.

For column testing, a borosilicate tube with a diameter of 1.5 cm and a column height of 20 cm was used. The ATB is charged into the column and wool is employed as an adsorbent bed. Figure 1 shows the descriptive experimental setup of the column experiment. A specified amount of ATB adsorbent was packed in the column (with desired bed depths) and wetted by distilled water prior to adsorption experiment. The fluoride solution was pumped continuously into the column down flow using a peristaltic pump. The solution was pumped through a column onto ATB adsorbent at different volumetric flow rate and samples were collected at 30-min interval. The fluoride level was measured using a using a spectrophotometer (Hitach-2000, Twinsburg, OH, USA) set to 570 nm.

For greater precision, fluoride analysis was performed in triplicates, and the average figures were recorded. Equations (1) and (2) were used to obtain the % removal and adsorption capacity of ATB, respectively, where *Qe* = adsorption capacity (mg/g), *V* = volume of solution in liter (L), *Co* = initial concentration (mg/L), *Ct* = fluoride concentration at equilibrium (mg/L), *m* = mass of adsorbent (g), and *R* = removal efficiency (%).

(1) $$\%\;Removal=\frac{\left(Co-Ct\right)}{Co}\times 100$$

(2) $$Qe=\frac{V}{m}\left(Co-Ct\right)$$

### 2.5. Design of Experimental Using Central Composite Design

The central composite design (CCD) was employed to optimize the adsorption conditions to obtain the maximum amount of F removal using the Design-Expert 7 (DX7, 7.1.5 provided by State-Ease company in Newcastle, England software program) [19,23]. The benefits of CCD are seen in its usage in sequential experimentation [24,25,26]. The goal of this design experiment is to find conditions for a maximum amount of % removal and/or adsorption capacity with optimized initial fluoride concentration, flow rate, and bed depth. Based on the results found from the previous study of the authors [15,27]; the workable range of the independent variables identified as initial influent fluoride concentration (designated as A or ‘X1′, (2–20 mg/L)), flow rate (designated as B or ‘X2′, (6–20 l/min)), and bed depth (designated as C or ‘X3′, (2–10 cm)) are identified as shown in Table 1.

The following is a representation (Equation (3)) of the relationship between appropriate response and predictor variable:

(3) $$Y=f\left(X1,X2,X3\ldots \ldots \ldots \ldots \ldots Xn\;\right)+\in$$

And *Y* stands for response, *X* denotes the predictor variables, *n* denotes number of factors under investigation, and ∈ denotes the sampling errors. The accompanying equation (Equation (4)) was used to calculate the coded values of process variables:

(4) $$Coded\;Value=xi=\frac{Xi-Xo}{∆X}\left(i=1,2,3,4\ldots \ldots ,K\right)$$

where *xi* is the coded variable, *Xi* is the actual value of an independent variable, *Xo* is the actual value of the independent variable at the center point and Δ*X* is the step change value of an independent variable. A total of 20 experimental runs were defined by the CCD design model of which six of the experimental runs were replicates at the center point of the experiment. Based on this design, the number of the experimental run chosen is suitable for the assessment of quadratic response surface and to generate a second-degree polynomial model, which is used to optimize an adsorption process using a small number of experimental runs. The design was tested at 0.05 level of significance. The relation between the independent and the response variables was assumed to be quadratic, and its general form of expression is given in Equation (5).

(5) $$Y=b_{0}+b_{1}X_{1}+b_{2}X_{2}+b_{3}X_{3\;}+b_{12}X_{1}X_{2}+b_{13}X_{1}X_{3}+b_{23}X_{2}X_{3}+b_{11}X_{1}^{2}+b_{22}X_{2}^{2}+b_{33}X_{3}^{2}$$

where *Y* is the response (% fluoride removal or adsorption capacity), *b*_0_ is offset term, *X*_1_, *X*_2_, and *X*_3_ are the independent variables, *b*_1_, *b*_2_, and *b*_3_ are linear terms, *b*_12_, *b*_13_ and *b*_23_ are interaction terms, and *b*_11_, *b*_22_, and *b*_33_ are quadratic terms.

## 3. Results and Discussions

### 3.1. Characterization of Acid-Treated Bentonite Adsorbent (ATB)

To observe the surface property (its moisture content (%), apparent density (g/cm^3^), pH, Cation-exchange capacity (CEC) meq/100 g, pHpzc, surface area (m^2^/g), and instrumental characterization such as XRD, SEM, EdX and FT-IR analysis results were reported in our earlier paper [13].

### 3.2. Statistical Analysis

Implementing central composite design with RSM process for optimization comprises four main steps [14]: (i) Generate a statistical experimental plan based on the independent variables and execute the experiment according to the plan. (ii) Propose a mathematical model according to the responses of the experimental results and elaborate the result of analysis of variance. (iii) Check the accuracy of the model through diagnostic plots (iv) Execute response analysis of the model and predict optimal conditions, and confirm the model through running an experiment.

#### Model Fitting and Analysis of Variance (ANOVA), Quadratic Model Equations, and Selected Model Diagnostic Test

Single and interaction effects of the independent factors on the % fluoride removal and fluoride adsorption capacity were studied by employing the CCD with RSM [19]. The CCD is frequently used for modeling and optimization, and it requires minimal number of experiments. In general, the CCD is made up of three types of runs: factorial runs, axial runs, and center runs [28,29]. A total of 20 experimental run accordingly were performed and the actual and predicted value of the responses along with the experimental conditions are summarized in Table 2.

### 3.3. Analysis of Variance (ANOVA)

The effect of main and international effects factors on % fluoride ion (R%) and adsorption capacity (mg/g) were analyzed by performing the analysis of variance (ANOVA) at 0.05 level of significance. The result of the analysis of variance (ANOVA) for the response surface quadratic model both for responses are given in terms of F and *p*-value (Table 3). The main and interaction effects of each factor with *p*-values < 0.05 are considered as having significant effect on the responses.

As the value of F-values or Sum of Squares rises, so is the relevance of that variable to significantly impact the % fluoride removal or adsorption capacity. The three factors are designated as initial fluoride concentration (A), flow rate (X_2_), and bed depth (C). It was observed that A, B, C, AC, A^2^, B^2^, and C^2^ impart significant effect on % fluoride removal. From the table it can be observed that only the interaction of initial concentration and bed depth has significant effect on fluoride removal. The “Lack of Fit F-value” for the % fluoride removal model is not high indicating the good fitness of the model. A significant “Lack of Fit F-value” has a 5.33% chance of being caused by noise in the case of fluoride removal. Furthermore, the adsorption capacity is significantly affected by A, B, C, A^2^, B^2^, and C^2^. The “Lack of Fit F-value” for both % fluoride removal and adsorption capacity is not high indicating the good fitness of the model. All for adsorption capacity, all interaction effects are insignificant. Therefore, the insignificant interactions will not be included in the model equation. The experimental data were analyzed, and the quadratic model was suggested to best fit for both % fluoride removal and fluoride adsorption capacity. To know whether the quadratic model is significant or not, use a sequential model sum of squares was applied. For instance, for % fluoride removal, according to model summary statistics, the quadratic model had the highest adjusted R^2^ 0.981 and projected R^2^ 0.9356 values for % fluoride removal. The 0.9356 “Pred R-Squared” and the 0.9810 “Adj R-Squared” values are in reasonable agreement since the rule of thumb for the adjusted R-squared basically plateaus when insignificant terms are added to the model, and the predicted R-squared will decrease when there are too many insignificant terms [30]. Furthermore, a smaller coefficient of variation (CV = 5.47%) suggests that the experiments were done with more precision and reliability [31]. Consequently, the quadratic model was chosen to describe the relationship. Using the results of the experiment, a regression model linking the response (Y) to the variables (X) relationship in terms of actual significant factors is expressed in Equations (6) and (7).

Fluoride removal = −93.73425 + 7.26 × Initial Fluoride Concentration + 12.41 × Flow Rate + 12.57 × Bed Depth + 0.69 × Initial Fluoride Concentration × Bed Depth − 0.97 × Initial Fluoride Concentration − 0.36 × Flow Rate − 0.92 × Bed Depth(6)

Adsorption Capacity = −4.56 + 0.28 × Initial Fluoride Concentration + 0.46 × Flow Rate + 0.39 × Bed Depth −0.024 × Initial Fluoride Concentration − 0.012 × Flow Rate − 0.018 × Bed Depth(7)

**Figure 2:**
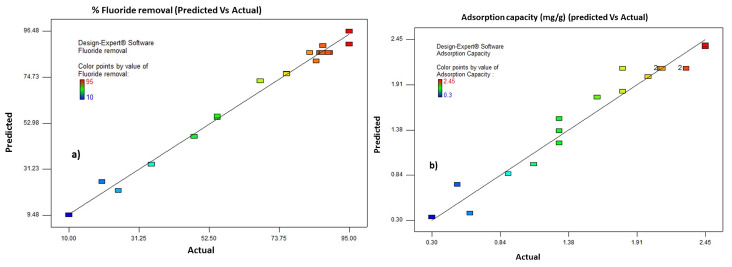
Correlation of actual and predicted values for (**a**) removal efficiency (R) and (**b**) Fluoride adsorption capacity (*Qe*).

The fitness and suitability of the developed regression models to the experimental results were further analyzed by the coefficients of determination (R^2^). An R^2^ value of 0.985 is reported for % fluoride removal model. An R^2^ value of 0.985 means that the model can explain 98.5% of the variation in % fluoride removal, and only 0.15% was as a result of chance. In addition to this, the predicted R^2^ (R^2^pre = 0.949) is in a reasonable agreement with the adjusted R^2^ (R^2^adj = 0.978) implying the high correlation between the actual and predicted values of % fluoride removal. The adequate precision value measures the signal to noise ratio and, a ratio greater than 4 is always desirable. In the case of % fluoride removal model an adequate precision value of 34.32 is obtained that indicates an adequate signal. Hence, this model can be used to navigate the design space. Similarly, for the adsorption capacity (mg/g) developed quadratic model the coefficient of determination (R^2^ = 0.927) indicates an excellent fit, meaning that the fitted model explained about 92.7% of the total variation in the adsorption capacity (mg/g). In addition to this, the difference between the adjusted R^2^ (R^2^adj = 0.893) and that of predicted R^2^ (R^2^pre = 0.817) is in reasonable agreement, which means high correlations existed between the actual and predicted value of % removal of fluoride. Finally, the adequate precision of the model is 15.7 hence, the suggested quadratic model is a good fit to the experimental data and can be used to navigate the design space. The model diagnostic test was performed using the predicted and actual values of for both responses as is shown in Figure 2a shows the predicted vs. actual value of the % fluoride removal response. All data points are either near to or laid on the y = x line, which indicates that the model is well fitted. Similarly, Figure 2b shows the predicted vs. actual values of adsorption capacities. Here again, all the data points lay near to the y = x line, which indicates the variability of the data is well explained by the generated model.

### 3.4. Effect of Operating Condition on the Adosrption Performance of ATB

**Figure 3:**
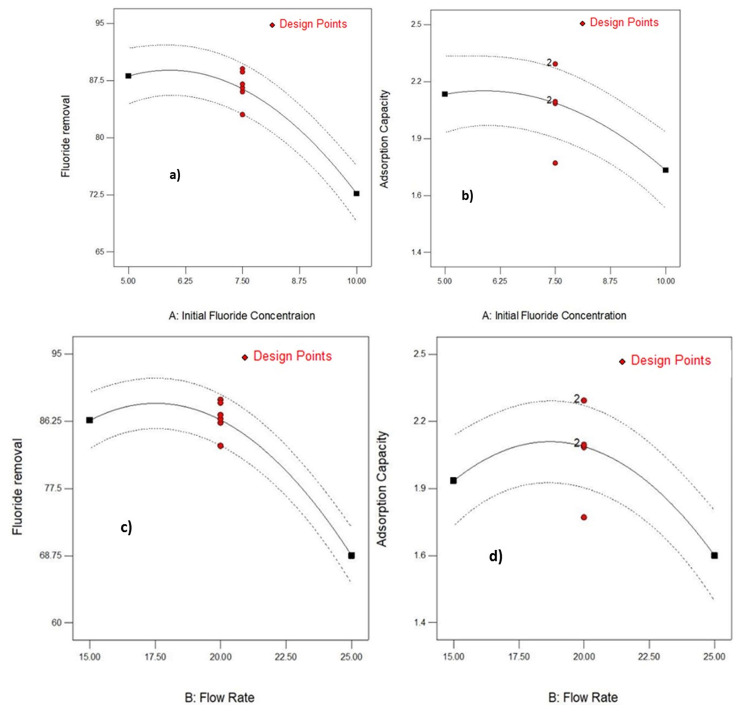

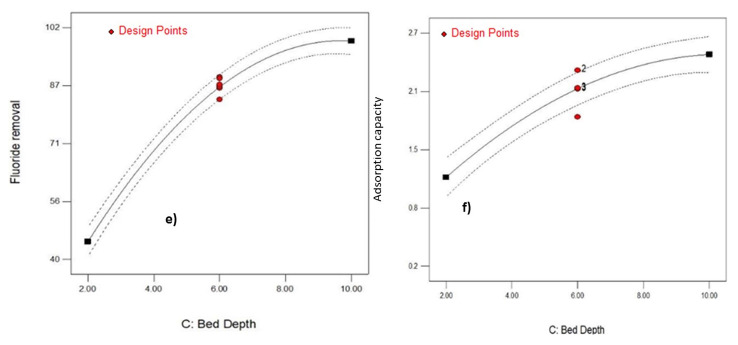
Effect of initial concentration, flow rate and bed depth on % fluoride removal and adsorption capacity (mg/g). (**a**) Varying initial Fluoride concentration on Fluoride removal; (**b**) Varying initial Fluoride concentration on adsorption capacity; (**c**) Varrying initial Flow rate on Fluoride removal; (**d**) Varying initial Flow Rate on Adsorption capacity; (**e**) Varying initial bed depth on Fluoride removal; (**f**) Varying initial bed depth on Adsorption capacity.

Figure 3 shows the singular effect of operating parameters on removal efficiency and adsorption capacity. It was observed that the three parameters have significant singular effect on removal efficiency and in any of the factors reasonably increases the removal efficiency. As shown in Figure 3e,f, the increase in bed depth increased % removal and adsorption capacity. Fluoride removal efficiency decreased from 92.5% to 70.2% with the increase of flow rate from 15 to 25 mL/min, respectively as shown in Figure 3c,d. Varying the initial fluoride concentration revealed (Figure 3a,b) very small effect on percent removal of fluoride and adsorption capacity. It was also observed that adsorption capacity is significantly increased with increase in bed depth whereas increase initial concentration results in relatively very small increase in adsorption capacity. The lines and red dots in Figure 3 indicates contour plots with average data points for column operational conditions. And also, the number 2 on the lines represent the level of response (i.e., adsorption capacity in mg/g) of the adsorbent.

### 3.5. Interaction Effect of Process Variables

**Figure 4:**
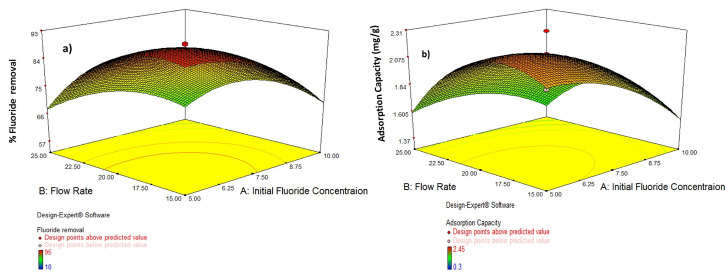

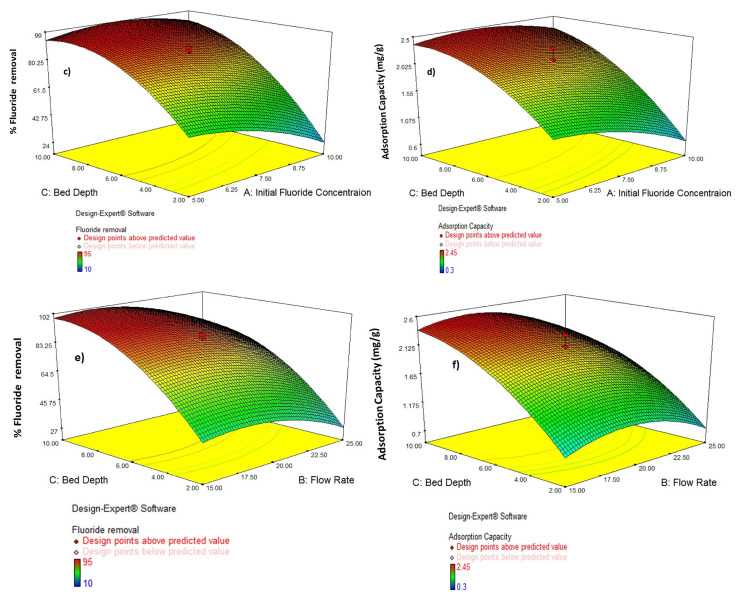
D surface plots for interaction effect of operating conditions on Fluoride removal efficiency (R) and adsorption capacity (mg/g) (**a**) Flow rate and initial fluoride concentration on Fluoride removal; (**b**) flow rate and initial fluoride concentration adsorption capacity and (**c**) adsorbent bed depth and initial fluoride concentration on fluoride removal (**d**) bed depth and initial fluoride concentration on Adsorption capacity (**e**) bed depth and flow rate on Fluoride removal and (**f**) bed depth and flow rate on adsorption capacity.

To evaluate the interaction effect of the three independent parameters on the adsorption productivity through fixed-bed column operations, several experiments with 1 cm to 13 cm of adsorbent packed-bed depth at flow rate of 12, 15, 20, 25 mL/min and initial fluoride concentrations 3, 5, 7.5, 10, 12 mg/L were conducted. To present the regression model output, graphical explanation using 3D response surface plots were shown for the optimization of fluoride adsorption on to acid-treated bentonite clay through fixed-bed column. The influences of the three different process variables (initial concentration, volumetric flow rate, and adsorbent bed depth) on removal efficiency (R) and fluoride adsorption capacity (*Qe*) as responses plot were determined. Figure 4a demonstrates that at lower levels, the combined effects of flow rate and initial fluoride concentrations have a considerably greater combined influence on removal efficiency. It was discovered that the influence of flow rate is greater than that of initial fluoride concentration, and that lowering any of these two parameters improves removal efficiency. The combined effect of flow rate and initial fluoride concentration on adsorption capacity is shown in Figure 4b. The combined effect of the two variables was found to be mostly controlled by flow rate, with the starting fluoride content having very little influence. It was also discovered that as the flow rate is increased, the adsorption capacity decreases. In addition, therefore, within the experimental range, the % fluoride removal dropped as both the starting fluoride concentration and the flow rate grew. The reality that almost all adsorbents have such a limited number of binding sites that become saturated at a particular concentration may explain this phenomenon. The binding sites on the adsorbent surface became more quickly saturated as the incoming fluoride concentration increased, ensuing in an earlier breakthrough. Additionally, with increased flow rates, the fluoride solution’s retention time in the column reduced, resulting in fluoride molecules not having enough time to grab binding sites on the surface of the adsorbent or penetrate the adsorbent pores, exiting the column before equilibrium was reached. Initial concentration and bed depth have a substantial combined influence on removal efficiency, as seen in Figure 4c,d, with bed depth having the biggest effect, but initial concentration has a decent effect as well. At higher levels of the two variables, the total effect was shown to be larger. It was also discovered that raising any of the parameters improves the efficiency of removal. Figure 4 depicts the combined effect of the two variables on adsorption capacity. It was discovered that at higher starting concentrations, increasing bed depth considerably reduces adsorption capacity, but at lower initial concentrations, increasing bed depth greatly increases adsorption capacity. This tendency can be explained by the fact that increasing bed height increases the number of binding sites available for the adsorption process. Since this is because of the fluoride molecules had more time to contact the adsorbent. The increased number of active sites available for adsorption might explain the first rise in fluoride ion adsorption. The adhering potential is high at the beginning phase, when the adsorbent surface is empty, and therefore sorption occurs at a faster rate. However, due to the saturation of active sites, the subsequent adsorption rate was sluggish, and equilibrium was only established over time [32]. Figure 4e,f shows the interactive effect of flow rate and bed height on % fluoride removal for dynamic adsorption of fluoride by ATB in a 3D plot. The % fluoride removal was significantly influenced by the interaction of flow rate and bed height. The percentage fluoride removed dropped as the flow rate rose, but it increased as the bed height grew. This trend may be explained by the fluoride molecules’ residence duration in the column as well as the presence of binding sites.

### 3.6. Optimization Using the Desirability Function

**Figure 5:**
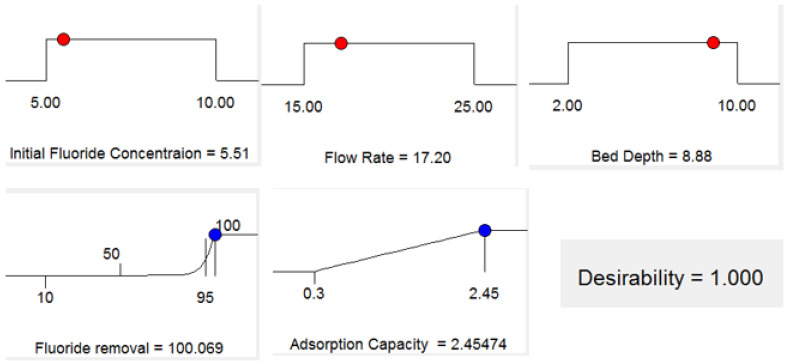
Desirability ramp for optimization (R = 100% and Desirability = 1.00).

It was examined the intended objective for each variable and the related response for numerical optimization [33]. Each objective was given a weight to change the form of its own desirability function. The initial fluoride concentration, flow rate, adsorbent packed-bed depth, adsorption capacity, and fluoride removal are all things we want to achieve. In this study, the input variables were given specific ranged values, whereas the response was designed to achieve a maximum as shown in Table 4. A desired value for each input element and response can be selected via numerical optimization. The input variables in this study were given precise values, and the response was designed to attain a maximum. The obtained findings are consistent with the experimental data, which show that the optimal parameters for fluoride removal are pH 7.2, bed depth of 8.88 cm, flow rate of 17.2 mL/min, and an initial fluoride content of 5.51 mg/L. At an initial fluoride concentration of 5.51 mg/L, a flow rate of 17.2 mL/min, and an adsorbent packed-bed depth of 8.88 cm with desirability of 1.0, the maximum achievable fluoride adsorption capacity was 2.46 mg/g (Figure 5). Under identical circumstances at pH = 7.2, the optimum conditions were verified three times (*n* = 3) (Table 5). The confirmatory experiment showed (Table 5) a fluoride removal efficiency of 100% under optimal conditions compared with the fluoride removal percent of 99.05% obtained by the model. This indicates the suitability and accuracy of the model.

### 3.7. Model Validation

Table 6 compares experimental data to anticipated values under optimal operating conditions. According to the table, the percentage error of experimental against anticipated value for adsorption capacity and removal efficiency is 2.43% and 1.00%, respectively. As a result, the models and optimal operating conditions created for the factors are valid and relevant in forecasting the response variables.

### 3.8. Comparison of Fluoride Adsorption Capacities between ATB and Different Clay-Based Adsorbents under Batch and Column Operations

The adsorption capacity of the adsorbent (ATB) was compared with different clay based-materials for fluoride adsorption as stated in Table 7 and it has relatively good result through packed fixed bed column experimentations.

## 4. Conclusions

The adoption of an experimental design using RSM strategy allows for the speedy testing of a broad experimental domain for the optimization of acid-treated bentonite adsorbent for fluoride adsorption capability. Under optimized condition, up to 99.05% fluoride was removed from effluents using ATB adsorbent with initial fluoride concentrations of 5.51 mg/L, flow rate of 17.2 mL/min, bed depth of 8.88 cm with a 1.00 desirability. Results suggest that ATB adsorbent has potential for fluoride adsorption. All the R^2^ values indicate that the models match the experimental data well. As far as we know, no acid-treated bentonite clay has been used as an adsorbent in the RSM method using continuously flowing fixed-bed column. This study shows that ATB are a potential adsorbent for the removal of fluoride ions from aqueous solution due to their high adsorption capacity and, more importantly, the fact that it is naturally and abundantly accessible at a reasonable cost.

## Figures and Tables

**Table 1:** Design experimentation variables and levels.

Name	Units	Type	Low	High
Initial influent fluoride Concentration (A)	mg/L	Factor	2	20
Flow rate (B)	l/min	Factor	6	20
Bed depth (C)	cm	Factor	2	10
Fluoride removal (R)	%	Response		
Adsorption capacity (Q)	mg/g	Response		

**Table 2:** Experimental runs and their predicted responses.

Category	Run	Factors			R%			*Qe* (mg/g)		
		A	B	C	Observed	Anticipated	Residual	Observed	Anticipated	Residual
Factorial points (8 runs)	3	10	15	10	87	61.901	−25.099	2.11	2.10825	−0.00175
	5	10	25	2	10	−37.349	−47.349	0.3	0.33295	0.03295
	14	5	15	2	55	41.31	−13.69	1.3	1.21145	−0.08855
	15	10	15	2	25	−6.975	−31.975	0.6	0.38445	−0.21555
	16	5	15	10	95	82.706	−12.294	2.45	2.35525	−0.09475
	17	5	25	10	68	49.786	−18.214	1.6	1.75375	0.15375
	19	5	25	2	35	9.886	−25.114	0.9	0.83995	−0.06005
	20	10	25	10	76	30.031	−45.969	1.8	1.82675	0.02675
Central point (5 runs)	2	7.5	20	6	86.5	58.322	−28.178	2.11	2.096175	−0.01383
	6	7.5	20	6	87	58.322	−28.678	2.11	2.096175	−0.01383
	11	7.5	20	6	88.6	58.322	−30.278	2.3	2.096175	−0.20383
	13	7.5	20	6	89	58.322	−30.678	1.3	2.096175	0.796175
	1	7.5	20	6	87	58.322	−28.678	2.1	2.096175	−0.00383
Axial points (6)	8	7.5	12	6	76	60.9836	−15.0164	1.3	1.572735	0.272735
	4	3	20	6	85	69.5	−15.5	2	1.9596	−0.0404
	7	12	20	6	55	8.264	−46.736	1.3	1.281	−0.019
	9	7.5	20	1	20	1.5795	−18.4205	0.5	0.816925	0.316925
	12	7.5	20	13		61.3215	61.3215	2.45	2.358325	−0.09168
	18	7.5	25	6		33.6485	33.6485	1.1	1.626425	0.526425

**Table 3:** Analysis of variance values of experimental design for % fluoride removal and adsorption capacity.

	% Fluoride Removal		Adsorption Capacity (q)	
Source of Variance	F-Value	*p*-Value Prob > F	F-Value	*p*-Value Prob > F
Model	110.29	<0.0001	23.84	<0.0001
A	59.62	<0.0001	13.1	<0.0047
B	77.32	<0.0001	9.23	0.0125
C	645.56	<0.0001	137.25	<0.0001
AB	4.04	0.0723 *	1.34	0.2744 *
AC	27.69	0.0004	4.39	0.0625 *
BC	0.082	0.7800 *	0.69	0.4262 *
A^2^	38.63	<0.0001	0.824	<0.0166
B^2^	83.39	<0.0001	35.63	<0.0001
C^2^	167.63	<0.0001	23.89	0.0006
Lack of fit	4.88	0.0533 *	1.38	0.3961 *

* Not significant model terms.

**Table 4:** Constraints for desirability analysis selected.

Name	Goal	Lower	Upper			
		Limit	Limit	Weight	Weight	Importance
Initial Fluoride Concentration	is in range	5	10	1	1	2
Flow Rate	is in range	15	25	1	1	3
Bed Depth	is in range	2	10	1	1	3
Fluoride Removal	maximize	50	100	10	1	3
Adsorption Capacity	maximize	0.3	2.45	1	1	3

**Table 5:** Optimization solutions provided by the model.

Number	Initial Fluoride Concentration	Flow Rate	Bed Depth	Fluoride Removal	Adsorption Capacity	Desirability	
1	6.84	18.22	8.59	100.505	2.46218	1	
2	7.17	18	8.67	100.513	2.45742	1	
3	5.51	17.2	8.88	100.069	2.45474	1	Selected
4	5.92	17.68	8.75	100.548	2.46739	1	
5	6.62	17.01	8.78	101.153	2.46102	1	
6	5.87	17.25	9.11	100.762	2.47643	1	
7	6.83	18.38	9.98	100.797	2.51664	1	
8	7.36	18.85	9.15	100.321	2.47696	1	
9	6.9	16.46	9.86	101.19	2.48363	1	
10	7.92	16.91	9.67	100.408	2.45459	1	
11	7.81	16.65	9.81	100.504	2.45688	1	
12	7.21	16.71	9.18	101.107	2.45836	1	
13	7.94	16.75	9.82	100.305	2.45293	1	
14	7.61	18.92	9.73	100.282	2.49188	1	
15	5.81	16.62	9.06	100.661	2.45847	1	
16	7.52	18.47	9.66	100.755	2.49698	1	
17	7.08	18.29	9.73	101.104	2.5106	1	
18	7.18	17.21	9.4	101.391	2.48524	1	
19	6.06	18.46	8.91	100.239	2.47605	1	
20	6.74	16.43	9.74	101.228	2.48135	1	
21	7.63	16.68	9.89	100.795	2.47035	1	
22	6.12	18.26	9.7	100.351	2.50193	1	
23	8.19	17.5	9.84	100.019	2.4607	1	
24	6.4	16.53	8.83	101.059	2.45055	1	
25	6.8	18.35	9.86	100.924	2.51484	1	
26	5.71	17.94	8.98	100.142	2.47162	1	
27	6.67	18	9.04	101.159	2.48933	1	
28	7.39	16.8	9.54	101.136	2.47189	1	
29	7.12	17.55	9.11	101.255	2.48051	1	
30	5.8	16.75	9.09	100.657	2.46263	1	
31	6.01	17.44	8.97	100.89	2.47735	1	
32	7.88	18.57	9.7	100.251	2.48316	1	
33	6.04	17.7	8.98	100.821	2.48062	1	
34	6.31	16.68	9.31	101.251	2.47734	1	
35	6.76	19.15	9.5	100.194	2.49312	1	
36	5.51	16.65	9.2	100.072	2.45363	1	
37	6.04	16.48	9.12	100.927	2.46062	1	
38	6.34	17.6	9.33	101.24	2.49749	1	
39	7.51	17.05	9.53	101.083	2.47487	1	

**Table 6:** Model validation.

Flowrate (mL/min)	Bed Depth (cm)	Concentrations (mg/L)	Experimental		Theoretical		Percentage Error	
			qe, (mg/g)	R (%)	qe, (mg/g)	R (%)	qe, (mg/g)	R (%)
17.2	8.88	5.51	2.2	99.05	2.46	100	2.43	1

**Table 7:** Bentonite based adsorbent were compared by their adsorptive capacity.

Adsorbent	Mode of Operation	Adsorption Capacity (mg/g)	References
Fired Clay Pots	Batch	1.6	[5]
Granular Acid-Treated Bentonite (GHB)	Batch and Column	0.094	[10]
Acid Activated Red Mud (Powdered)	Batch	5.06	[34]
Tunisian Kaolinite	Batch	1.48	[35]
Diatomite Modified with Aluminum Hydroxide	Batch	1.67	[36]
Mn2+-Modified Bentonite Clay	Batch	0.08	[37]
Al3+-modified Bentonite Clay	Batch	5.7	[38]
Magnesium Incorporated Bentonite Clay	Batch	2.26	[39]
Dicarboxylic Acid (Malic Acid (A)), Metal Ion Decorated Bentonite Clay (BC) Modified with Chitosan (CS)	Batch	9.87	[40]
Acid-Treated Bentonite (ATB)	Column	2.46	This study

## Data Availability

Data used for the study will be provided by corresponding authors upon the request.

## Sample Availability

Samples of the compounds are available from the authors.
